# Wind energy development and wildlife conservation in Lithuania: A mapping tool for conflict assessment

**DOI:** 10.1371/journal.pone.0227735

**Published:** 2020-01-15

**Authors:** Rasa Morkūnė, Mantas Marčiukaitis, Viačeslav Jurkin, Giedrius Gecevičius, Julius Morkūnas, Liutauras Raudonikis, Antanas Markevičius, Aleksas Narščius, Zita R. Gasiūnaitė

**Affiliations:** 1 Marine Research Institute, Klaipeda University, Klaipėda, Lithuania; 2 Lithuanian Energy Institute, Kaunas, Lithuania; 3 Coastal Research and Planning Institute, Klaipėda, Lithuania; 4 University of Applied Sciences, Kaunas, Lithuania; 5 Lithuanian ornithological society, Vilnius, Lithuania; Wuhan University, CHINA

## Abstract

The paper presents a mapping tool aiming to identify and minimise potential conflicts between onshore wind energy development and wildlife conservation in Lithuania. It merges current information on the distribution, conservation status and sensitivity of birds and bats to wind power with an integrated evaluation of wind resources (modelled wind speed), special planning status and technical perspectives of wind energy development. The paper includes assessment of the selected wildlife species which were described as sensitive to wind power (69 breeding and 43 migratory bird species and 17 bat species bats in the country). Used species level information allowed the precise identification of sensitive territories and might be used to mitigate negative wind farm effects using special measures based on species behavior. Finally, we delivered overlaps as possible conflicts among the most promising wind farm areas and the areas with high sensitivity in relation to bird and bat distribution. These overlaps point to the required attention and relevant decisions that are needed to ensure sustainable development of wind energy throughout the country. We suggest this tool for initial determination of appropriate areas for wind energy development in the country and as supplement to Environmental Impact Assessment.

## Introduction

With low greenhouse gas emissions compared to fossil fuels, wind energy provides an important contribution to renewable energy (for review see [1]). Wind energy is being developed in many European countries, including Lithuania. The National Energy Independence Strategy of the Republic of Lithuania (hereinafter–Strategy) envisages that by 2030 the country will have achieved an installed wind power capacity limit of 1250 MW. The generation of electricity and heat based on environmentally-friendly technologies will enable Lithuania to reduce greenhouse gas emissions by 40% and 60% by 2030 and 2040 respectively and achieve an 80% reduction of greenhouse gas emissions by 2050 [2]. According to the 2017 data, operating wind farms produce 12% of total net production (1.3 TWh). Therefore, the implementation of the Strategy will expand the area used for wind farms by almost 2.5 times, while harmonization of economic, technical, social and environmental interests may become a challenging task.

The economic evaluation of wind energy development highly depends on wind resources, and thus wind speed modelling is essential to provide valuable spatial information for the development of this sector. Technical limitations to connect to grid however might prevent development of wind energy or concentrate wind farms into particular regions where grid connection is more applicable. Special planning at the municipality level might also result in the development of wind energy at sites which are the most suitable in terms of inhabitants and/or other anthropogenic or conservation activities [3; 4; 5].

Development of wind farms has negative aspects associated with the death of birds and bats that collide with the turbines and other energy related structures, as well as habitat loss or changes, disturbance and barrier effects [6; 7]. The possibility of collision of birds or bats with wind turbines is considered to be smaller than the risks faced from other anthropogenic activities [8], but the wildlife mortality at wind farms is different from other causes of mortality with respect to which species and age groups are affected and, therefore, the risk of potential long-term effects of wind energy on wildlife should not be neglected [9]. It is a concern that bird and bat fatalities could become a serious issue if wind farms are deployed in sensitive areas, potentially resulting in a reduction of wildlife. In this case, even relatively small increases in mortality rates may be significant for populations of rare species, especially large, long-lived species with generally low annual productivity [10]. As the most efficient way to minimize the risk to wildlife is to avoid installation of wind farms in areas where the risk is potentially high, sensitivity mapping has been performed for some countries or the most risky areas. Species of conservation concern were mapped for the territory of the United Kingdom [11; 12], migratory soaring birds were focused upon for the Red Sea flyway [13] and risk areas for migratory non-soaring birds were modelled across Switzerland [14]. Many species data were used for the sensitivity mapping in the Netherlands and Belgium [15; 16]. It has been agreed that appropriate planning, collection of wildlife data in areas where information is limited and avoidance of high-risk sites are prerequisites for the effective minimization of the negative effects of wind energy development on wildlife and the taintless development of wind farms [9; 11; 17]. Therefore, even after wind turbines are installed, options to minimize impacts should be tailored to species at a particular site [17].

Sensitivity mapping should help to identify sites that potentially are sensitive for wildlife and avoid environmentally sensitive area. However, these need to be compensated against other demands, such as wind resources, connectivity to the electricity grid, visibility in the landscape, human activities, other energy resources. Multiple-criteria practice that requires a balance of economic, technological, societal, and environmental demands within a spatial context might be a required solution for sustainable development of wind energy [17]. During initial planning, developers should be encouraged to use available tools and information to identify the most suitable areas for development of wind energy.

There are available mapping tools designed to help the development of usage of energy sources in different countries, for example to identify potential energy resource areas and corridors for different energy resources in U.S. [18]. An extended study for spatial planning of wind energy in New York State includes a number of economic, sociologic and environmental factors, while only an official dataset for *Important Bird Areas* was used in that study, while any other wildlife data were not considered [19]. It is important to note that peculiarities of multiple-criteria decision-making tools should be decided at local scale, because pools of available information strongly limit contents of mapping tools.

In order to meet ambitious renewable energy targets, the expansion of wind farms can become difficult to control even under the official surveillance of stakeholders. In Lithuania, only a small number of developing wind farms have fulfilled the relevant environmental assessment procedures with wildlife surveys at the preconstruction stage and thereafter followed requirements for post-construction monitoring. Therefore, there is a need for a tool that could facilitate the future selection of potential wind farm sites or the demand to collect more detailed wildlife information. At the same time, the selection of lower conflict areas might ensure the smoother process of wind farm development, transparency and equal opportunities to all investors.

This paper presents a tool that is designed to minimize the potential conflicts between wind farm development and wildlife conservation. It merges current information on the distribution, conservation status and sensitivity of breeding and migratory birds and bats to wind farm development together with social-economic and technical perspectives of wind energy development in Lithuania. Overlaps between the most promising wind farm areas and mapped sensitivity in relation to bird and bat distribution reveals a scale of conflicts. As such, conflicts of varying strengths require attention and relevant decision-making to ensure sustainable wind energy development throughout Lithuania.

## Methodology

### Study area

This study aimed to cover the whole terrestrial area of Lithuania (65286 km^2^), located in north-eastern Europe. Forests cover 33.7% of the country, agricultural fields 47.5%, meadows and natural pastures 5% and urban territories 3.7% of the area (2016 data from the National Land Service under the Ministry of Agriculture [20]). 15.4% of the terrestrial country area has been designated as protected territories, including Natura 2000 sites (13%) [21].

**Fig 1:**
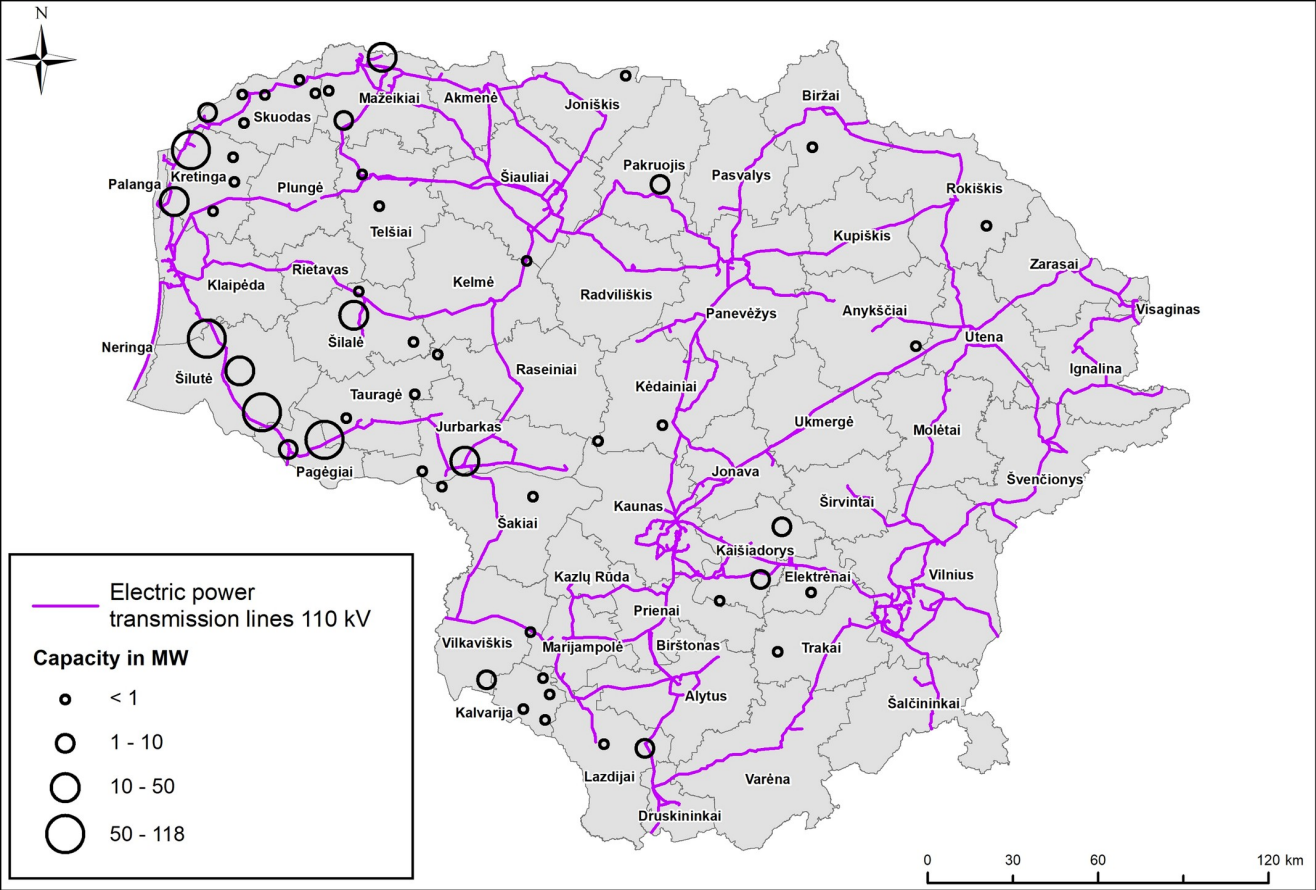
Operating wind turbines and power transmission lines in 2017 in Lithuania. Size of circles indicates energy capacity in MW; capacities of overlapping wind farms were summed for the presentation on this map.

The country is divided into administrative units (municipalities, districts), which each differ in terms of their preparation of special plans for wind energy development. At the beginning of 2017, 20 wind farms with a total installed power capacity of 521 MW operated in the country (including only farms with >350 kW). The installed power capacity at separate wind farms varied in the range 6–73.5 MW (Fig 1), while the number of wind turbines ranged from 3 to 30. The hub height of the installed wind turbines is from 78 to 134 meters and the rotor diameters vary between 70 and 120 meters.

Based on wind measurement data from meteorological stations, the annual mean wind speed at 50 m above ground varies from 3.5 to 6.42 m/s through the municipalities of Lithuania (calculated according to the data of the Lithuanian Hydrometeorological Service). This shows that wind resources differ significantly between the municipalities, therefore their precise estimation is important for power production and economically effective wind energy development.

### Evaluation of conflict between wind energy development and wildlife

**Fig 2:**
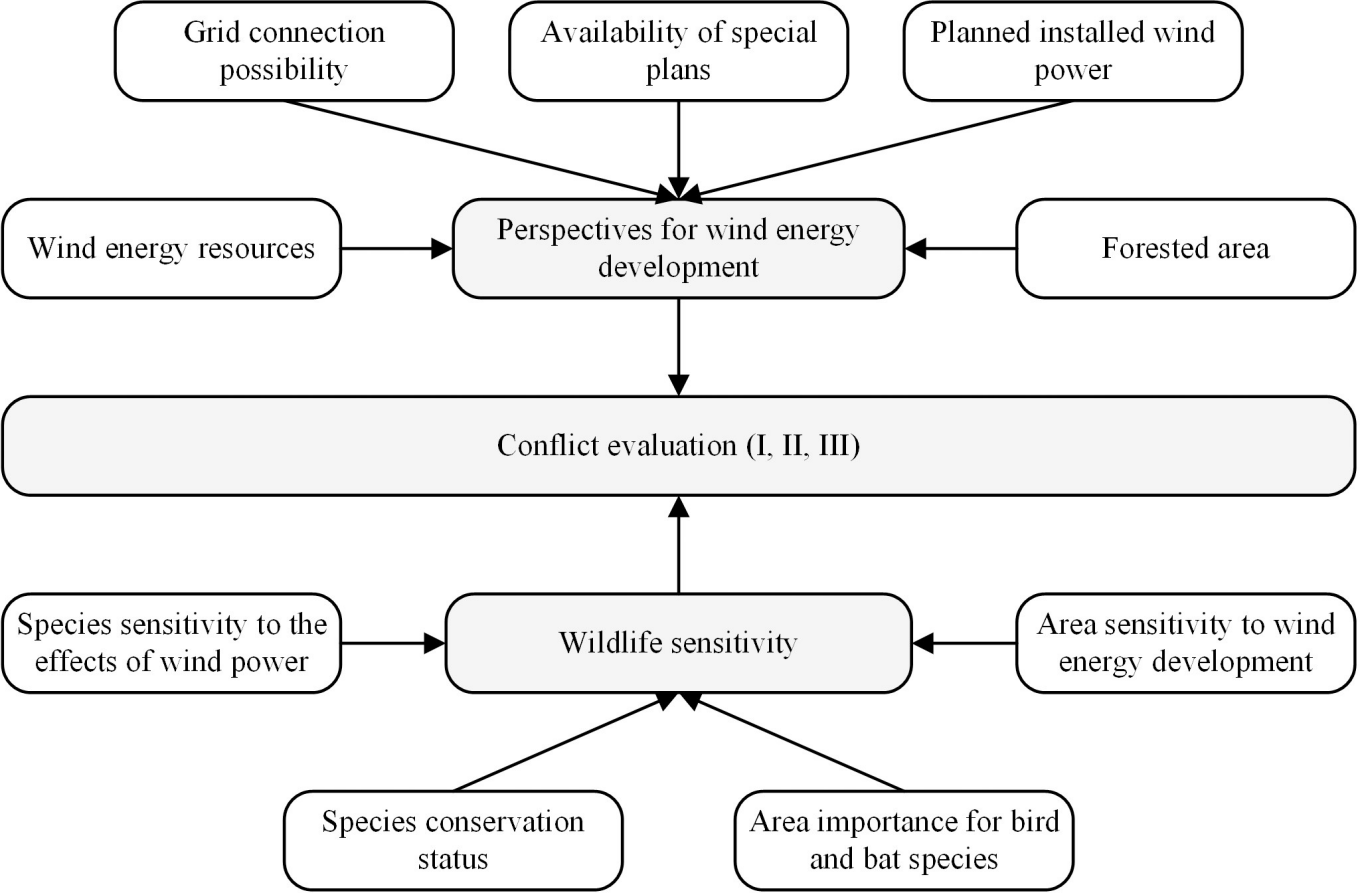
Logical scheme of estimations used to evaluate conflicts between wind power development and wildlife conservation in this study.

An integrated assessment of wind energy development and wildlife sensitivity was performed using a sequence of procedures (Fig 2). The first part of the conflict evaluation consisted of an assessment of the perspectives of wind energy development which was complemented by wind resource modelling and local area parameters. The second part of the evaluation was devoted to wildlife sensitivity assessment by using species traits and the importance of areas for birds and bats. The final assessment of conflict between the perspectives for wind development and wildlife sensitivity is presented as the final result of this work.

### Assessment of wind resources in municipalities

The wind resources in all municipalities (except Palanga, Neringa, Visaginas where wind development is not possible) were described by the mean wind power density at a height of 50 m, which was derived from wind measurement data at hourly intervals from meteorological stations over a single year (2016).

Wind is measured at a height of 10 m at the meteorological stations, therefore the following equation was used for the evaluation of wind speed at a height of 50 m [22].

$$u_{50}=u_{10}\frac{\text{ln}\frac{z_{50}}{z_{0}}}{\text{ln}\frac{z_{10}}{z_{0}}}$$ (1)

where: *u*_*10*_ – wind speed at 10 m height (m/s)*; z*_*50*_ – height above ground level for the estimated wind speed (m)*; z*_*10*_ – height of wind speed measurements (m), z_0_ – roughness length (m). Roughness length was estimated approximately from aerial photos of the measurement site surroundings. Additionally, ArcGIS Desktop 10 applications as well as spatial data sets GDR10LT and GDR50LT were used for terrain and topographical conditions modelling all over the country. There are only 18 meteorological stations in Lithuania, so wind measurement data for the missing regions were taken from neighboring municipalities where data was available.

Weibull distribution parameters were used for the assessment of mean power density, calculated by the Maximum Likelihood Estimate (MLE) method using WindPRO 3.0 and WAsP 9 software.

### Perspectives for wind energy development

Five criteria for assessing the prospects of wind energy development in municipalities were selected (also indicated in Table 1 with values for each municipality)

- Wind energy resources (W/m^2^);
- Possibility of connection to the grid (MW available, data of the TSO, Litgrid AB);
- Special plans for the development of the wind farms;
- Forecasted total installed power of the planned wind farms in a municipality;
- Percentage of forests in a municipality.

**Table 1:** Input parameter and results of the integrated assessment of wind energy development and conflicts with wildlife in different municipalities.

Municipality	Area, km^2^	Information about wind farm development								Total estimated area of municipality in the context of wildlife, %	Area of different level of conflict between priority of wind development and wildlife, %		
		Installed wind power (> 350 kW), MW	Wind energy density (at 50 m), W/m^2^	Capacity of power transmission 100 kV lines, MW	Special plan for wind turbines development	The planned capacity of wind farms, MW	Forest area, %	Overall estimation (as I from Eq 3), points	Priority score (1 is high and 3 is low)		High	Medium	Low
Akmene	844	0	132	70	+	63	32.4	0.7	**2**	63.1	22.9	12.5	27.7
Alytus	1443	0	148	150	-	-	25.1	0.75	**2**	68.9	34.1	15.0	19.7
Anyksciai	1764	7.5	120	60	+	-	32.7	0.6	**2**	55.2	33.1	9.6	12.5
Birstonas	122	0	167	0	-	-	47.1	0.5	**3**	72.0	0.0	64.3	7.7
Birzai	1476	0	90	100	-	0.03	27.2	0.85	**2**	86.9	39.8	27.1	20.1
Druskininkai	453	0	66	70	-	-	69.1	0.3	**3**	52.6	0.0	31.7	20.9
Elektrenai	509	0	167	70	-	-	32.8	0.75	**2**	66.3	41.7	15.3	9.3
Ignalina	1441	0	89	100	-	-	36.4	0.65	**2**	61.6	40.8	13.7	7.0
Jonava	943	0	137	130	+	41	41.8	0.95	**2**	56.5	20.9	8.6	27.0
Joniskis	1152	0	91	70	-	-	20.2	0.5	**3**	62.9	0.0	20.4	42.6
Jurbarkas	1506	24	157	60	+	89.8	38.1	1.15	**1**	62.4	28.2	11.8	22.4
Kaišiadorys	1088	6	167	125	+	48.93	32.8	1.3	**1**	79.6	45.3	20.8	13.5
Kalvarija	440	0	190	0	+	14.4	14.2	1.65	**1**	91.4	67.4	7.5	16.5
Kaunas	1652	0	167	295	-	51.27	32.5	1.35	**1**	81.5	45.8	17.4	18.3
Kazlu Ruda	555	0	190	150	-	-	60	0.9	**2**	22.0	5.1	2.6	14.3
Kedainiai	1677	0	90	110	-	120.1	25.1	0.95	**2**	68.5	37.6	8.7	22.2
Kelme	1705	0	91	60	+	100.8	31.8	0.8	**2**	53.0	22.0	8.6	22.4
Klaipeda	1438	0	342	60	-	69.19	26.4	1.3	**1**	87.5	43.3	16.4	27.7
Kretinga	989	106.56	342	60	+	39.61	34.9	1.4	**1**	80.2	25.0	13.4	41.8
Kupiskis	1080	0	90	100	-	-	29.5	0.75	**2**	61.4	39.0	7.4	15.1
Lazdijai	1306	6	148	150	-	1.55	35.9	0.75	**2**	48.3	33.0	3.1	12.3
Marijampole	755	0	190	50	-	2	15.4	1.45	**1**	59.4	36.3	11.6	11.5
Mazeikiai	1220	45.6	132	10	+	22.2	29.5	0.8	**2**	58.6	21.2	10.7	26.7
Moletai	1367	0	66	70	-	-	31.5	0.4	**3**	62.9	0.0	41.8	21.1
Pagegiai	90	77.5	342	50	+	100.83	17.4	1.6	**1**	73.5	56.3	8.6	8.6
Pakruojis	535	6	91	50	+	22	19.8	0.8	**2**	79.9	27.3	20.2	32.3
Panevezys	1315	0	120	140	-	0.05	34.9	0.75	**2**	67.6	34.8	12.8	20.0
Pasvalys	79	0	90	100	-	-	17	0.75	**2**	89.2	19.8	38.5	30.9
Plunge	2227	0	149	60	-	-	36.5	0.4	**3**	79.2	0.0	46.8	32.3
Prienai	1289	0	167	150	-	5	27.7	1.2	**1**	56.7	24.3	13.0	19.3
Radviliskis	1105	0	91	50	-	-	25.9	0.5	**3**	54.2	0.0	25.3	28.9
Raseiniai	1032	0	157	60	-	68.5	23.8	0.95	**2**	58.4	25.0	10.3	23.1
Rietavas	1634	0	220	60	-	-	54.1	0.65	**2**	56.4	12.4	9.8	34.2
Rokiskis	1573	0	90	100	-	15.15	29.3	0.85	**2**	68.6	34.8	9.5	24.2
Skuodas	586	0	132	0	-	8.2	10.9	0.35	**3**	64.9	0.0	12.6	52.3
Sakiai	1806	0	190	50	+	88.5	23	1.25	**1**	58.0	31.0	10.9	16.0
Salcininkai	911	0	66	0	-	-	48.3	0.15	**3**	50.8	0.0	25.8	25.0
Siauliai	1453	0	91	70	-	-	34.9	0.4	**3**	61.2	0.0	30.7	30.5
Silale	1493	14.6	220	60	+	213.13	28.7	1.35	**1**	53.5	19.0	9.0	25.4
Silute	1888	72.5	342	60	+	151.41	42.1	1.6	**1**	85.5	61.4	13.1	11.0
Sirvintos	1188	0	137	70	-	-	33.3	0.4	**3**	64.3	0.0	19.6	44.7
Svencionys	1714	0	77	100	-	-	60.4	0.55	**0**	57.1	0.0	48.9	8.2
Taurage	905	44.9	142	0	-	125.02	38.7	0.35	**3**	42.3	0.0	20.5	21.8
Telsiai	1692	0	149	140	-	21	35.6	0.75	**2**	55.1	16.0	10.2	28.9
Trakai	1179	0	111	70	-	-	49.9	0.3	**3**	58.9	0.0	32.2	26.8
Ukmerge	1438	0	137	60	-	-	32.6	0.4	**3**	56.2	0.0	34.7	21.5
Utena	1207	0	66	300	-	-	33.7	0.9	**2**	57.0	26.6	15.3	15.2
Varena	1395	0	66	70	-	-	68.8	0.3	**3**	52.3	0.0	43.7	8.6
Vilkaviskis	1230	0	190	150	-	37.25	11	1.2	**1**	54.6	9.5	15.1	29.9
Vilnius	2216	0	111	70	-	-	41.7	0.4	**3**	46.7	0.0	21.4	25.2
Zarasai	1262	0	89	70	-	19.25	37.6	0.5	**3**	72.6	0.0	39.6	32.9

Each of these criteria had a non-dimensional value of 1 to 3 and a weight factor according to the (2) formula. Non-negative value was obtained by dividing the range of variations of each criterion into three equal parts. Municipalities were divided into three groups according to the sum of points calculated using formula:

I=A×0.35+B×0.25+C×0.20+D×0.10−E×0.10 (2)

where:

A – parameter of wind energy resources, in a range from 60 to 342 W/m^2^ (Table 1);
B – free grid capacity to connect to 110 kV network, in a range from 0 to 295 MW (Table 1);
C – preparation status of a special plan for wind farm development with values 1 ("prepared") or 0 ("not prepared"). A prepared special plan facilitates the development of a wind farm and saves at least one year of project development, thus it contributes to the current state of municipality attractiveness to potential developers (Table 1);
D – total projected power of the planned wind farms in the municipality, in a range from 0 to 213 MW (Table 1);
E – area of municipality covered with forests, in range from 11 to 69% (official land cover data of 2016; Table 1).

The results of the calculation were divided into three groups according to the sum of points (I priority >1.1, II priority– 0.6–1.1, III priority <0.6). The municipalities with the highest point numbers were assumed to have the most favorable conditions for wind energy development.

Wind energy development prospects in the municipalities were demostrated using the Geographical Information System (GIS) Esri ArcGIS for Desktop version 10.4.1 and GRPK Spatial data set of (geo) reference base cadastre [23].

### Bird and bat sensitivity

The estimations of bird and bat sensitivity were based on the distribution of selected priority species, their sensitivity to different effects of wind farm development and operation and on their national and international conservation status.

The priority species selected for the mapping were those known as displaying a particular risk in relation to wind farms during the breeding or migration seasons in the country. In total, 69 breeding and 43 migratory bird species and all 17 registered bat species were included in further analysis, while species with little evidence of adverse wind energy effects were not included.

Overall sensitivity scores for each bird and bat species were estimated as

A=D×(B+C) (3)

where

**A** – overall score of sensitivity to wind energy development, which was based on a three-level scale of sensitivity rating (Table 2).
**B** – conservation score (Table 2) based on importance to national conservation (categories of Lithuanian Red Book, LRB) for birds and bats and international conservation (IUCN list) for birds additionally. If the conservation categories for the birds differed between the LRB and IUCN, the score was set according to the LRB.
**C** – score of species sensitivity (Table 2) to suspected effects of wind energy development as (a) collisions, b) disturbance, c) barrier effects, or d) habitat loss/ change which were set using a three-level scale of sensitivity ratings according to Tables 2 and 3 for birds and Table 4 for bats. For birds, a comparison of the total scores of sensitivity to wind power effects for breeders and migrants of the same species was performed using Wilcoxon signed rank sum test. For bats, the sensitivity of different species was rated from 1 to 3 depending on preferred flight height, the possibility of hunting close to structures or further away from them and whether the species is already known for collisions at wind turbine farms (according [24], Table 4).

**Table 2:** Description of wildlife sensitivity and scores for the final evaluation.

Parameter		Birds		Bats
		Breeders	Migrants	
A	Overall sensitivity score	(High) >12		(High) >20
		(Medium) between 7 and 12		(Medium) between 9 and 20
		(Low/ unknown) between 1 and 6		(Low/ unknown) between 2 and 8
B	Conservation score	(3)—categories of 0 (Extinct) or 1 (Endangered) in LRB and CR or EN in IUCN		(3) - 0 or 1 in LRB
		(2)—categories of 2 (Vulnerable) or 3 (Rare) in LRB and VU or NT in IUCN		(2) - 2 or 3 in LRB
		(1)—categories of 4 (Intermediate) or 5 (Restored) in LRB and LC in IUCN		(1) - 4 or 5 in LRB
C	Sensitivity to wind energy development	(2)–high		(3)–high
		(1)–medium		(2)–medium
		(0)–low		(1)–low/ unknown
D	Local relative abundance	(3) >0.5% of overall country population	(3) observed abundance is higher than significant number for a species	Number of bat species at 1-km square resolution, which was based on actual observations
		(2) between 0.5%-0.1% of overall country population	(2) observed abundance is between minimal and significant number of individuals	
		(1) <0.1% of overall country population	(1) observed abundance is lower than minimal number of individuals	

Numbers in brackets show scores. LRB–Lithuanian Red Book, IUCN—International Union for Conservation of Nature and categories as CR—critically endangered, EN—endangered, NT—near threatened, LC—least concern species.

**Table 3:** Characteristics of breeding bird species for the estimation of sensitivity to wind power.

No	Bird order	Bird species	Buffer zone in meters	Score of conservation status	Score of sensitivity to effects of wind power during breeding period					National breeding population		
					Collision	Disturbance	Barrier	Habitat change/ loss	Summed score of sensitivity	Number of breeding pairs	0.5% of breeding population	0.1% of breeding population
1	Accipitriformes	*Accipiter gentilis*	500	2	2	0	1	0	3	500	3	1
2		*Accipiter nisus*	500	1	2	0	1	0	3	4000	20	4
3		*Aquila chrysaetos*	2000	3	2	0	1	0	3	1	1	1
4		*Buteo buteo*	1000	1	2	0	1	0	3	6000	30	6
5		*Circus aeruginosus*	1000	1	2	0	1	0	3	3500	18	4
6		*Circus cyaneus*	1000	3	2	0	1	0	3	1	1	1
7		*Circus pygargus*	1000	3	2	0	1	0	3	300	2	1
8		*Clanga clanga*	2000	3	2	0	1	0	3	1	1	1
9		*Clanga pomarina*	2000	2	2	0	1	0	3	1900	10	2
10		*Haliaeetus albicilla*	2000	2	2	0	1	0	3	120	1	1
11		*Milvus migrans*	1000	3	2	0	1	0	3	40	1	1
12		*Milvus milvus*	1000	3	2	0	1	0	3	20	1	1
13		*Pandion haliaetus*	1000	3	2	0	1	0	3	25	1	1
14		*Pernis apivorus*	1000	2	2	0	1	0	3	1000	5	1
15	Anseriformes	*Aythya ferina*	500	2	1	1	0	0	2	3000	15	3
16		*Anas clypeata*	500	2	1	1	0	0	2	200	1	1
17		*Anas penelope*	500	2	1	1	0	0	2	5	1	1
18		*Anas strepera*	500	3	1	1	0	0	2	250	2	1
19		*Anser anser*	500	2	1	1	0	0	2	200	1	1
20		*Cygnus cygnus*	500	2	1	1	0	0	2	300	2	1
21		*Mergus merganser*	500	2	1	1	0	0	2	1000	5	1
22	Caprimulgiformes	*Caprimulgus europaeus*	200	1	1	0	0	0	1	4000	20	4
23	Charadriiformes	*Calidris alpina*	500	3	2	0	0	0	2	5	1	1
24		*Chlidonias hybridus*	1000	3	2	0	1	0	3	50	1	1
25		*Chlidonias leucopterus*	1000	3	2	0	1	0	3	100	1	1
26		*Chlidonias niger*	1000	3	2	0	1	0	3	3000	15	3
27		*Gallinago gallinago*	500	1	1	0	0	0	1	10000	50	10
28		*Gallinago media*	500	3	2	0	0	0	2	100	1	1
29		*Larus argentatus*	1000	2	2	0	1	0	3	300	2	1
30		*Larus cachinnans*	1000	1	2	0	1	0	3	100	1	1
31		*Larus canus*	1000	1	2	0	1	0	3	300	2	1
32		*Larus minutus*	1000	2	2	0	1	0	3	50	1	1
33		*Larus ridibundus*	1000	1	2	0	1	0	3	30000	150	30
34		*Limosa limosa*	500	3	2	0	0	0	2	250	2	1
35		*Numenius arquata*	500	3	2	0	0	0	2	50	1	1
36		*Philomachus pugnax*	500	3	2	0	0	0	2	200	1	1
37		*Pluvialis apricaria*	500	3	2	0	0	0	2	40	1	1
38		*Sterna hirundo*	1000	2	2	0	1	0	3	2000	10	2
39		*Sternula albifrons*	1000	3	2	0	1	0	3	200	1	1
40		*Tringa glareola*	500	3	2	0	0	0	2	100	1	1
41		*Tringa totanus*	500	3	2	0	0	0	2	400	2	1
42		*Vanellus vanellus*	500	1	2	0	0	0	2	10000	50	10
43	Ciconiiformes	*Ardea alba*	500	2	2	1	0	0	3	50	1	1
44		*Ardea cinerea*	500	1	2	1	0	0	3	3000	15	3
45		*Ciconia ciconia*	500	1	2	1	0	0	3	20000	100	20
46		*Botaurus stellariss*	500	2	1	1	0	0	2	1500	8	2
47		*Ciconia nigra*	2000	3	2	1	0	1	4	600	3	1
48		*Ixobrychus minutus*	1000	2	1	1	0	0	2	30	1	1
49	Columbiformes	*Columba oenas*	500	2	2	0	0	0	2	500	3	1
50		*Columba palumbus*	100	1	0	0	0	0	0	60000	300	60
51		*Streptopelia turtur*	500	2	2	0	0	0	2	2000	10	2
52	Coraciiformes	*Coracias garrulus*	500	3	1	0	0	0	1	10	1	1
53	Falconiformes	*Falco columbarius*	500	3	2	0	0	0	2	5	1	1
54		*Falco peregrinus*	1000	3	2	0	0	0	2	1	1	1
55		*Falco subbuteo*	1000	2	2	0	1	0	3	700	4	1
56		*Falco tinnunculus*	1000	3	2	0	1	0	3	200	1	1
57	Galliformes	*Lyrurus tetrix*	1000	2	0	0	0	0	0	1500	8	2
58	Gaviiformes	*Gavia arctica*	500	3	1	1	0	0	2	7	1	1
59	Gruiformes	*Crex crex*	500	2	0	0	0	1	1	23000	115	23
60		*Grus grus*	2000	2	0	2	0	0	2	5000	25	5
61	Passeriformes	*Acrocephalus paludicola*	500	3	1	0	0	0	1	100	1	1
62		*Corvus frugilegus*	1000	2	2	0	1	0	3	30000	150	30
63		*Emberiza hortulana*	500	2	1	0	0	0	1	60	1	1
64		*Luscinia svecica*	500	2	1	0	0	0	1	200	1	1
65	Podicipediformes	*Podiceps auritus*	500	3	1	1	0	0	2	5	1	1
66		*Podiceps cristatus*	500	1	1	1	0	0	2	15000	75	15
67	Strigiformes	*Apus apus*	1000	3	1	0	0	0	1	20	1	1
68		*Asio flammeus*	500	3	1	0	0	0	1	30	1	1
69		*Asio otus*	500	1	1	0	0	0	1	3000	15	3

**Table 4:** Scores of conservation status and sensitivity to wind energy development for bats.

Bat species	Score of conservation status	Score of sensitivity to wind power (from 1 as low/ unknown to 3 as high)
*Myotis brandtii*	2	1
*Myotis myotis*	3	1
*Myotis dasycneme*	3	2
*Myotis nattereri*	2	1
*Myotis daubentonii*	1	1
*Myotis sp*.	1	1
*Vespertilio murinus*	2	3
*Barbastella barbastellus*	3	1
*Nyctalus noctula*	2	3
*Nyctalus leisleri*	2	2
*Plecotus austriacus*	1	1
*Plecotus auritus*	2	1
*Eptesicus serotinus*	2	3
*Eptesicus nilssoni*	2	3
*Pipistrellus pygmaeus*	2	3
*Pipistrellus pipistrellus*	2	3
*Pipistrellus nathusii*	1	3

**D for birds** – the local relative abundance in a particular area in comparison to the overall country population. For breeders, the local abundance was evaluated as being the percentage of the overall breeding population in the country and compared to 0.5 and 0.1% of it [25] (Tables 2 and 3). Areas for migratory species concentrations were estimated using minimal and significant numbers of individuals (Tables 3 and 5), which show the importance of a particular area to migratory bird concentrations (limits were set using local expert knowledge).

**Table 5:** Characteristics of migratory bird species for estimation of sensitivity to wind power.

No.	Bird order	Bird species	Buffer zone in meters	Score of conservation status	Score of sensitivity to effects of wind power during migration					Abundance at stopover sites (number of individuals)	
					Collision	Disturbance	Barrier	Habitat change/ loss	Summed score of sensitivity	Minimal abundance	Significant abundance
1	Accipitriformes	*Buteo buteo*	1000	1	2	0	1	0	3	10	15
2	Accipitriformes	*Haliaeetus albicilla*	2000	1	2	0	1	0	3	5	10
3	Accipitriformes	*Clanga pomarina*	2000	2	2	0	1	0	3	3	8
4	Anseriformes	*Aythya fuligula*	500	2	1	1	0	0	2	100	500
5	Anseriformes	*Anas penelope*	500	2	1	1	0	0	2	200	500
6	Anseriformes	*Anas platyrhynchos*	500	1	1	1	0	0	2	300	500
7	Anseriformes	*Anas strepera*	500	1	1	1	0	0	2	20	50
8	Anseriformes	*Anser albifrons*	500	1	1	1	0	0	2	500	1000
9	Anseriformes	*Anser anser*	500	1	1	1	0	0	2	10	40
10	Anseriformes	*Anser erythropus*	500	3	1	1	0	0	2	1	5
11	Anseriformes	*Anser fabalis*	500	1	1	1	0	0	2	300	1000
12	Anseriformes	*Branta leucopsis*	500	1	1	1	0	0	2	20	100
13	Anseriformes	*Bucephala clangula*	500	1	1	1	0	0	2	200	1000
14	Anseriformes	*Cygnus cygnus*	500	1	1	1	0	0	2	20	50
15	Anseriformes	*Cygnus columbianus*	500	3	1	1	0	0	2	10	20
16	Anseriformes	*Cygnus olor*	500	1	1	1	0	0	2	50	100
17	Anseriformes	*Melanitta fusca*	500	2	1	1	0	0	2	100	200
18	Anseriformes	*Mergus albellus*	500	1	1	1	0	0	2	20	50
19	Anseriformes	*Mergus merganser*	500	1	1	1	0	0	2	30	80
20	Ardeidae	*Ardea cinerea*	500	1	1	0	1	0	2	20	50
21	Charadriiformes	*Calidris alpina*	500	1	1	0	0	0	1	20	50
22	Charadriiformes	*Chlidonias hybridus*	1000	3	1	0	1	0	2	20	100
23	Charadriiformes	*Chlidonias leucopterus*	1000	3	1	0	1	0	2	20	100
24	Charadriiformes	*Chlidonias niger*	1000	2	1	0	1	0	2	20	50
25	Charadriiformes	*Gallinago gallinago*	500	1	1	0	0	0	1	30	50
26	Charadriiformes	*Larus cachinnans*	1000	1	1	0	1	0	2	50	100
27	Charadriiformes	*Larus canus*	1000	1	1	0	1	0	2	300	500
28	Charadriiformes	*Larus marinus*	1000	1	1	0	1	0	2	10	20
29	Charadriiformes	*Larus minutus*	1000	1	1	0	1	0	2	50	150
30	Charadriiformes	*Limosa limosa*	500	2	1	0	0	0	1	5	15
31	Charadriiformes	*Numenius arquata*	500	2	1	0	0	0	1	10	100
32	Charadriiformes	*Philomachus pugnax*	500	3	1	0	0	0	1	50	100
33	Charadriiformes	*Pluvialis apricaria*	500	1	1	0	0	0	1	100	500
34	Charadriiformes	*Vanellus vanellus*	500	2	1	0	0	0	1	100	500
35	Ciconiiformes	*Ciconia ciconia*	500	1	2	1	0	0	3	50	100
36	Ciconiiformes	*Ciconia nigra*	2000	2	2	1	0	1	4	4	10
37	Ciconiiformes	*Egretta alba*	500	1	2	1	0	0	3	50	100
38	Columbiformes	*Columba palumbus*	100	1	2	0	0	0	2	50	100
39	Gruiformes	*Fulica atra*	500	1	1	1	0	0	2	100	500
40	Gruiformes	*Grus grus*	2000	1	0	2	2	0	4	50	200
41	Passeriformes	*Corvus frugilegus*	1000	1	1	0	0	0	1	200	500
42	Pelecaniformes	*Phalacrocorax carbo*	500	1	1	0	0	0	1	200	500
43	Podicipedidae	*Podiceps cristatus*	500	1	1	1	0	0	2	20	50

**D for bats** – the number of bat species at a one-kilometer square resolution, which was based on actual observations (Table 2).

Territories where the sensitivity of birds and bats were not investigated are identified as “not estimated”. The identification of sensitive areas was based on actual bird and bat observation data, information about wildlife in designated Natura 2000 territories and 11 operating regional dumping sites. Actual bird (10360 points and 1103 polygons) and bat (7138 points and 45 polygons) data were collected by field observers between 2015–2017 [26]. Additional data of 3718 points, collected during 2010–2016, were gathered from [27].

In order to identify the sensitive areas for wind energy development, we analyzed information from all 559 Natura 2000 territories throughout Lithuania, which are designated for the protection of birds (84) and habitats (475). We used the Natura 2000 grid due to its clear focus on the conservation of certain species, while other protected areas have been designated for a broader spectrum of protected objects. In Lithuania, the majority of Natura 2000 sites overlap with other protected areas. In this study, all Natura 2000 territories were considered as highly sensitive territories. Additionally, considering the possible wind energy development effects on species of importance in particular Natura 2000 areas, buffer zones varying from 500 to 2000 meters (Tables 3 and 5) were additionally selected for 71 bird and 16 habitat areas.

The buffer zones (as disturbance-free zones) for birds were based mostly on feeding distance and home range for particular species (Tables 3 and 5). The buffer zones for all breeding bat species were set at 1000 meters, while for wintering places at 2000 meters. These buffer zones were estimated by local experts according to [9; 12; 25].

As open dumping sites attract high numbers of gulls, storks and other bird species, all 11 regional dumping sites were mapped as important areas. The 2000-meter buffer zone and known passage corridors, located between breeding or stopover areas and dumping sites, were considered to be of high sensitivity.

Calculations and a digital map of ranked bird and bat sensitivities to the development of wind energy in Lithuania at a 1-km square resolution were made using the Esri ArcGIS for Desktop version 10.4.1. Distribution data for each bird and bat species were mapped on separate layers–most of the data were vectorial, but the areas of Natura 2000 sites and regional dumps were polygonal. The buffers were added to the distribution data according to Tables 2 and 3 and sensitivity ratings were applied to these buffer areas. The final result was a map on wildlife sensitivity to wind power, developed using the Eq 3. More detailed results on http://corpi.lt/venbis/index.php/observation/maps

### Conflict evaluation matrix

An integrated assessment of wind energy development and wildlife sensitivity was performed using a matrix for conflict estimation (Table 6). The results at a municipality level were used to produce a map as final assessment with a scale of conflicts using Esri ArcGIS for Desktop (10.4.1 version). The percentages of areas of high, medium and low conflict zones were calculated for each municipality (Table 6).

**Table 6:** Matrix for conflict estimation among perspectives of wind energy development and wildlife sensitivity.

Priority for wind energy development	Wildlife sensitivity		
	High	Medium	Low
I (best)	High	Medium	Low
II (medium)	High	Medium	Low
III (low)	Medium	Low	Low

## Results

### Assessment of wind energy development through the country

**Fig 3:**
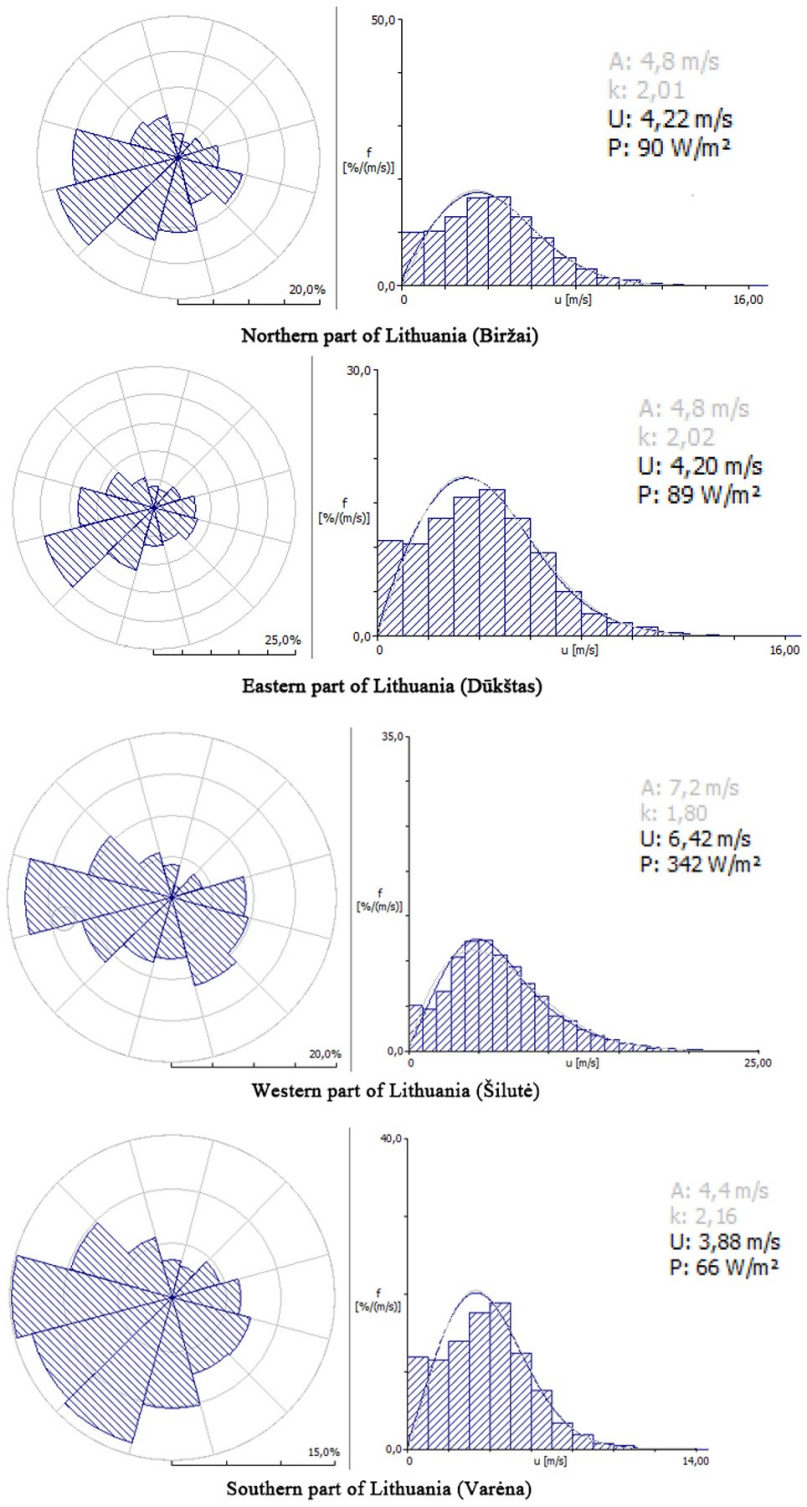
Wind direction and speed parameters in 2016 in different parts of Lithuania. A – scale parameter; k – shape parameter; U – wind speed (m/s), P – wind power density (W/m2).

Statistical analysis on the main Weibull function’s parameters in the different parts of Lithuania showed that the highest average wind speed of 6.42 m/s with a power density 342 W/m^2^ was in the western part of Lithuania (Fig 3). In comparison, the average wind speed estimated in the eastern part was 4.2 m/s with a power density of 89 W/m^2^. Northern and southern parts of the country presented an average wind speed of 4.22 m/s and 3.88 m/s respectively and the difference between these average wind speeds was not significant. However, the power density in the northern part (90 W/m^2^) was 27% higher than in the southern part (66 W/m^2^).

The distribution of operating wind farms and 110 kV power lines in Lithuania are shown in Fig 1 and Table 1. Small scale (<350 kW) stand-alone wind turbines and smaller wind farms (up to 6 MW) are connected to 35 kV voltage power lines.

**Fig 4:**
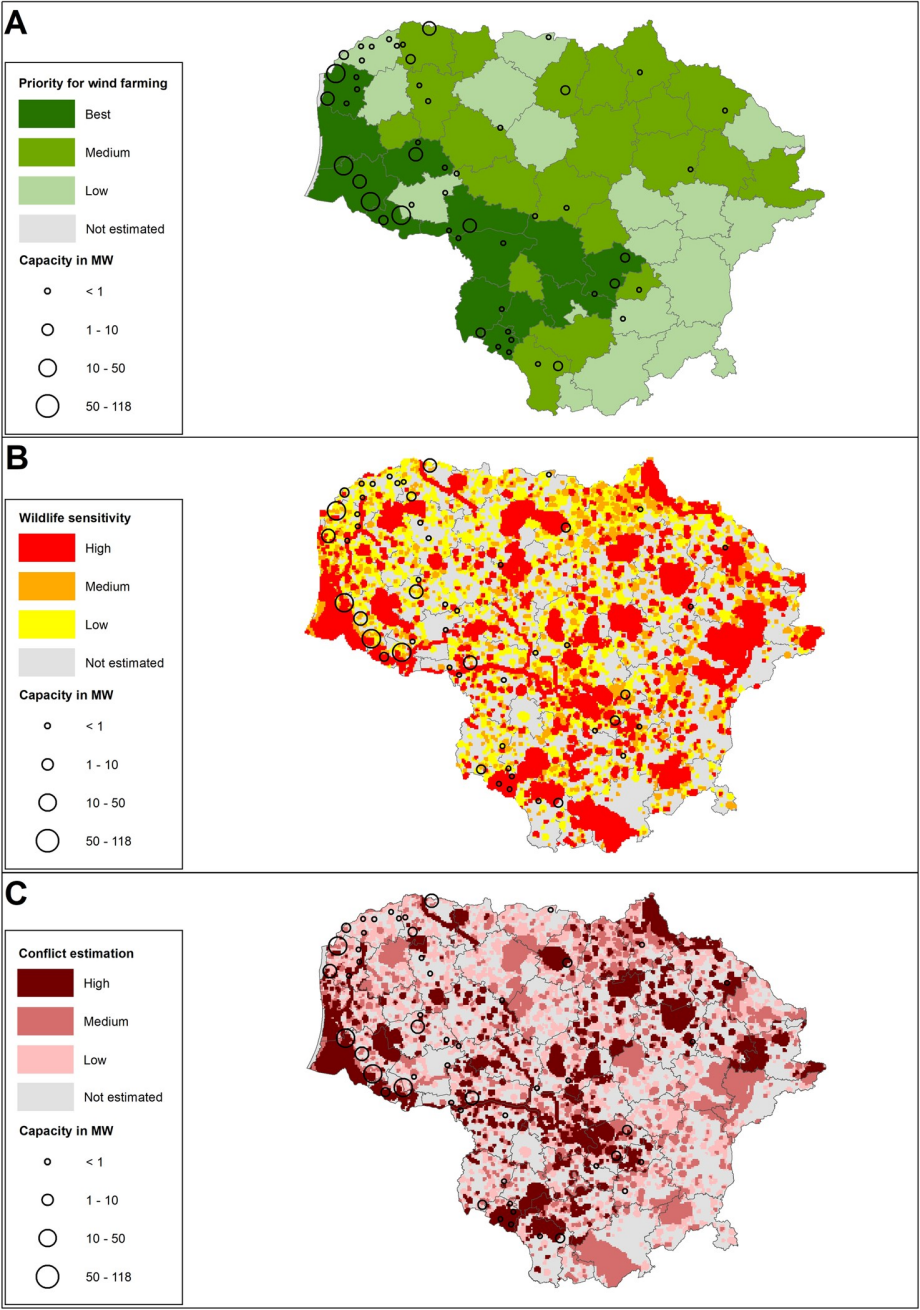
Perspectives of wind development (A), wildlife sensitivity to wind power (B), and assessment of their conflicts in Lithuania (C).

Results of the integrated assessment of wind energy development prospects in the municipalities are presented in Fig 4A and Table 1. Approximately 73.2% of installed wind power (>350 kW) was located in first priority zones, while 14.4% and 12.4% of the power was installed in second and third priority zones for wind power.

### Wildlife sensitivity to wind power

**Fig 5:**
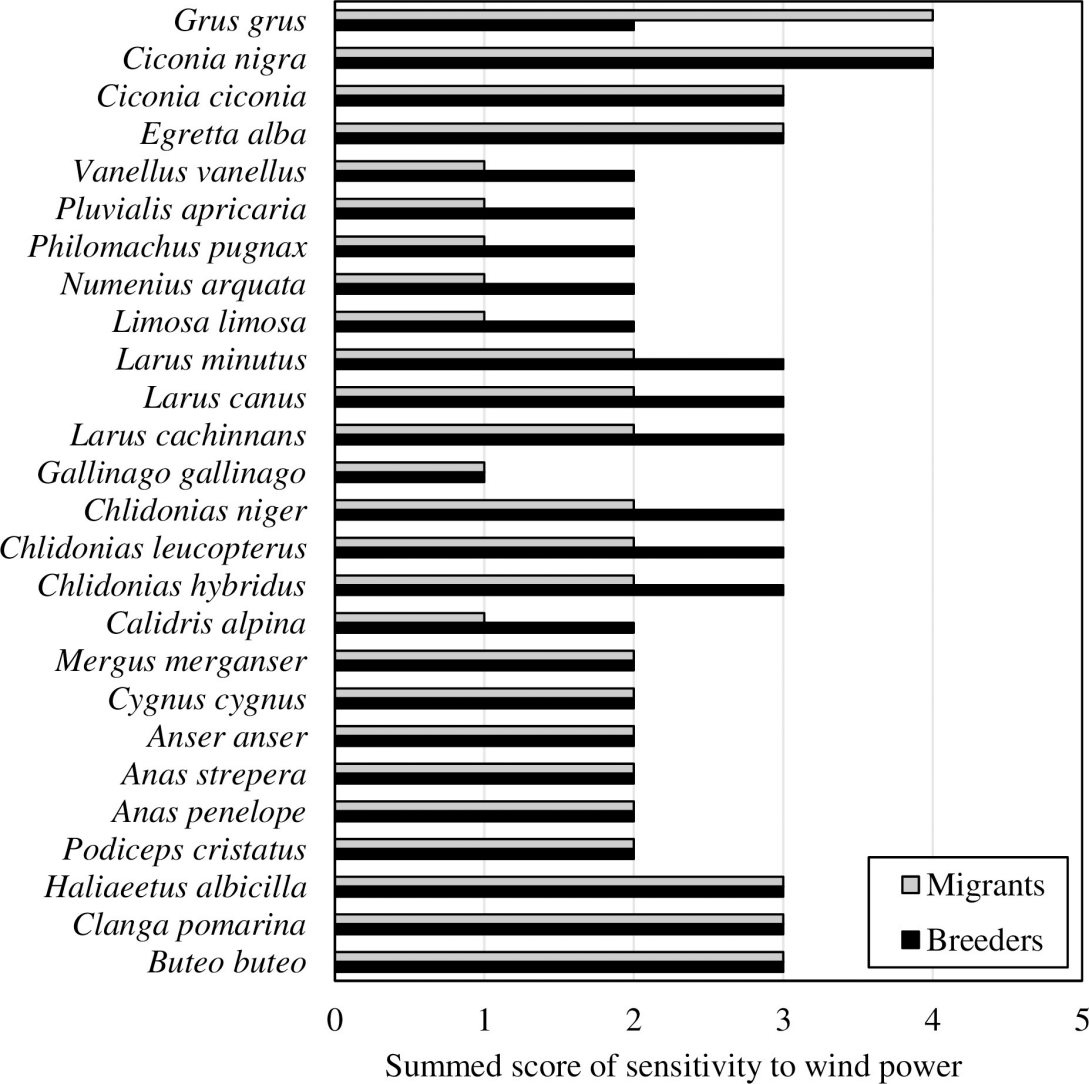
Cumulative sensitivity to wind power effects for species that are both breeders and migrants.

The total scores of sensitivity to wind power differed among the studied bird species as well among breeding and migrating birds of the same species. Breeding Black and White Storks, birds of prey and colonies of terns and gulls were considered to have the highest scores of sensitivity to the different effects of wind power among breeding bird species (Table 3). Storks, Great White Egret, White-tailed Eagle, Lesser Spotted Eagle, Common Buzzard and Common Crane had the highest scores during the migration period (Table 5). There was a significant difference in the total scores of sensitivity to wind power effects for breeders and migrants of the same species (Wilcoxon signed rank sum test, p<0.05). Common Crane appeared to be more sensitive to wind power during migration than during the breeding season. However, higher sensitivity scores were applied for breeding gulls, terns, and waders, but lower for migrants of the same species (Fig 5). Wildlife sensitivity was assessed for 64% of the country's territory, covering an area of 41.715 km^2^ (Fig 4B). Within this, an area amounting to 32% (21.111 km^2^) of the country's area was assessed as high sensitivity, while 19% (12.434 km^2^) as medium sensitivity. Wildlife in the western part of the country in the Curonian Lagoon region, as well as the municipalities of Varėna, Biržai and Anykščiai were characterized as highly sensitive to wind power (Fig 4B).

### Assessment of conflicts between wind energy development and wildlife conservation

Wind energy development and wildlife sensitivity were assessed and integrated, with the resulting conflict zones mapped in Fig 4C. Though the assessment of wind energy perspectives covered the entire territory of Lithuania, the wildlife was evaluated for only 64% of the country territory (Fig 4B); thus, the conflict areas were estimated on the same scale as wildlife assessment. At the final evaluation, the conflict areas in municipalities were distributed differently: high conflict areas occupied on average 21.2% (0 to 67.4% in different municipalities), medium– 19.6% (2.6 to 64.3%), low– 22.7% (from 7 to 52.3%) of the studied area in the municipalities.

Considering the installed wind power (by MW), 27% of installed wind power in 2017 was located in the high conflict zones, 26% in the medium conflict zones, while 47% of installed energy was located in low conflict zones.

## Discussion

In this study, we designed and applied a tool aiming to identify potential conflicts between perspective areas for wind farm development and wildlife conservation in Lithuania. Similar tools used in other countries include mostly information only about bird species, sometimes matching it to protected areas, using published data or expert knowledge [28]. Our tool merges current information on the distribution, conservation status and sensitivity of birds and bats to wind power together with integrated wind resource evaluation, special planning status and technical perspectives of wind energy development through the Lithuanian municipalities.

### Estimation of wind resources and potential for wind energy development

In this study, wind speed modelling was essential and provided valuable spatial information for the possibilities of wind farm development in Lithuania. Technical limitations were also assessed to determine where issues with connections to the national grid might prevent development of wind energy or might concentrate wind energy investments into particular regions where grid connection is more applicable. Also considered were regional special planning schemes that might promote wind energy development in sites which are most suitable in terms of inhabitants and/or other anthropogenic or conservation activities.

This study revealed that the highest average wind speed of 6.42 m/s with a power density 342 W/m^2^ was in the western part of Lithuania (Fig 3). These results confirmed the former findings of other authors and the results of other wind measurement campaigns that also found that the western part of Lithuania stands out as having the most attractive wind power resources. However, this region is also famous for its intensive bird migration and bird stopover sites [29] and this issue has already been a hindering factor for several wind power development projects. Thus, these issues must be taken seriously as the total wind power capacity expands in the nearest future.

The capacity of electricity transmission lines is also an important factor limiting wind farm development. In Lithuania, the localities of operating wind farms are highly determined by the locations of 110 kV power lines (Fig 1). However, Kalvarija municipality in the middle part of the country, which has no 110 kV power lines available, is within the first priority zone due to its relatively higher wind resources, lower proportions of forest area and the presence of a special plan.

Concerning large-scale wind turbine development, most zones of the first priority are already occupied (of the 13 high priority municipalities, seven do not have operating wind farms). Accounting for 21 municipalities located all around the country, the second priority zones may become important for further wind farm development. Third priority zones cover the larger part of the country (16 municipalities) and are less suitable for the development of wind energy due to unfavorable factors as large proportions of forestry or limited grid capacity. Even these questions can be solved, only the investments and payback time will not be so attractive.

Economically based wind farm development is directly related to the wind resource at the chosen sites. Considering landscape variations and surface roughness, wind speed can be significantly different in the same municipality. Therefore, as wind farm developers seek the most suitable sites within municipalities, wind resource modelling becomes an important method to identify them, even it requires additional scientific contributions (as an example, wind resource modelling for Kalvarija municipality is presented in Box 1).

**Box 1. An example of wind resource modelling in Kalvarija municipality.**

**Fig 6:**
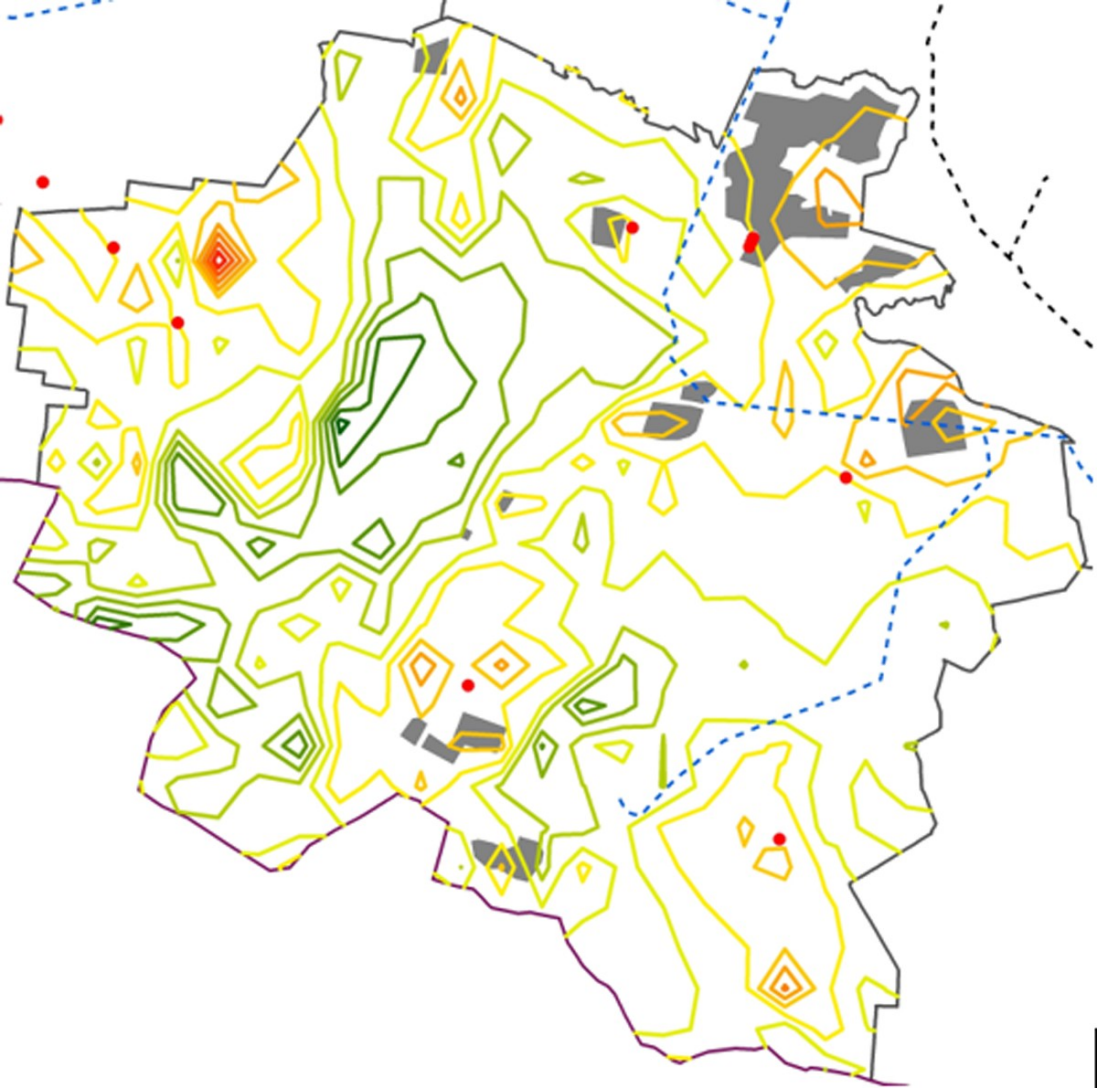
Wind energy development situation in Kalvarija municipality. Solid colored lines represent wind resources from the lowest (green) to the highest wind speed (red); grey polygons mark planned zones for wind energy development, blue dashed lines represent high voltage grid lines.

The situation of wind energy development in Kalvarija municipality is shown as an example of using distributions of planned zones for wind energy development according to the special plan for the municipality, with a modelled wind speed at 100 m height and available high voltage grid lines. The wind energy development zones in the special plans however were determined only based on the land use, with wind resources not taken into consideration. In Fig 6, the planned zones for wind farm development lay within areas of good wind resources, but there are many more areas with favorable wind resource than determined in the special plan. Even if the power grid is not available in some of the areas, the wind resource map can be a useful tool facilitating wind energy related spatial planning procedures in the future. Results of other municipalities are online on http://corpi.lt/venbis/index.php/home/ve

When wind resource calculations and solutions of special planning contradict each other, a final priority estimation may be problematic. As special planning procedures require approval from all land owners within a 2 km radius of each planned wind turbine (Klaipeda district), the final evaluation might not be favorable for investors or for further wind farm development. In general, this circumstance is considered as an obvious social hindrance and leads to less promising final estimations, but due to the methodology used in this study Klaipeda municipality was still classified as first priority area for wind power. because it has free grid capacity and wind resource there is among the highest in Lithuania.

### Mapping of wildlife sensitivity and conflicts with wind farm development

The wildlife sensitivity map (Fig 4B) was created using the available information on the distribution of sensitive bird species and bats in the country. It is a new approach for sensitivity assessments of wind farm development, as similar studies mostly took into account only a small number of the most sensitive bird species [11] or groups of species according to their taxonomy, habitats or behavior [17]. Most of published focus groups for the mapping were species of conservation concern for whole countries [11; 12], migratory soaring birds or modelled non-soaring birds in the most risky territories, including flyways [13; 14]. A high number of species (e.g. 105) or groups of bird species were mapped only for some areas in the Netherlands, Belgium and South Africa [15; 16; 30]. Thus, usually the greatest concern of environmental impact assessments has been that of species of high conservation importance and high sensitivity. However, having in mind that bird and bat species are differently susceptible to negative interactions with wind energy [8; 25], our study was based on information of a relatively large number of bird and bat species (Tables 3 and 5). Detailed species level information throughout the country allowed the precise identification of sensitive territories to wind energy development, according to species behavior analysis (e.g. buffer zones depended on species mobility). Moreover, this species level information might be used to mitigate negative wind farm effects using special measures based on species behavior.

Our tool provides spatial overlaps between sensitivity in relation to bird and bat distribution and their habitats in the most promising wind farm areas. The overlaps expressed possible conflicts in varying strengths between wind farm development and wildlife conservation and, at the same time, pointed out the required attention to ensure the sustainable development of wind energy through the country. Obviously, the areas with a high level of conflict require more attention from wind farm developers, environmentalists and decision makers than areas with medium or low conflict estimates. As reported in [9], this type of sensitivity tool not only helps to indicate areas of high conflict level with a greater probability of significant negative effects on wildlife, but also determine the necessity of special conditions and requirements for wind power installation. If wind farm developers decide to invest and install wind farms in a medium or high conflict level area, this decision potentially should require more detailed and longer-term information to assess the potential impacts and appropriate mitigation or compensation. In addition, estimates based on species level information might allow consideration of medium conflict areas for wind energy due to the possibility to ensure mitigation of the wind power effects for particular species. However, incompleteness of data in some territories and the absence of long-term monitoring in the country did not allow the separation between areas with low wildlife sensitivity to wind power and unknown status. Moreover, regular updating of sensitivity maps is necessary for the successful use of the tool in future.

The estimated high conflicts among perspectives of wind energy development and wildlife sensitivity mean very unlikely development of wind energy plants in particular areas. Otherwise, it is important to note that low conflict level designation for an area does not mean the absence of wildlife, but rather it could show a lower possibility (but not absence) of conflicts there. Furthermore, medium and especially low conflict areas or unstudied areas in the final maps require more wildlife surveys in the early stages of the decision making process. These limitations determine that the tool is not a substitute for site-specific Environmental Impact Assessment but rather a supplement, revealing a conceivable understanding of wildlife distribution, perspectives and limitations for wind energy development.
